# Exploring the Structural Transformation Mechanism of Chinese and Thailand Silk Fibroin Fibers and Formic-Acid Fabricated Silk Films

**DOI:** 10.3390/ijms19113309

**Published:** 2018-10-24

**Authors:** Qichun Liu, Fang Wang, Zhenggui Gu, Qingyu Ma, Xiao Hu

**Affiliations:** 1 Center of Analysis and Testing, Nanjing Normal University, Nanjing 210023, China; 40021@njnu.edu.cn; 2 School of Chemistry and Materials Science, Nanjing Normal University Jiangsu, Nanjing 210023, China; guzhenggui@njnu.edu.cn; 3 School of Physics and Technology, Nanjing Normal University, Nanjing 210023, China; maqingyu@njnu.edu.cn; 4 Department of Physics and Astronomy, Rowan University, Glassboro, NJ 08028, USA; 5 Department of Biomedical Engineering, Rowan University, Glassboro, NJ 08028, USA; 6 Department of Molecular and Cellular Biosciences, Rowan University, Glassboro, NJ 08028, USA

**Keywords:** silk fibroin, glass transition, DMA, FTIR, stress-strain

## Abstract

Silk fibroin (SF) is a protein polymer derived from insects, which has unique mechanical properties and tunable biodegradation rate due to its variable structures. Here, the variability of structural, thermal, and mechanical properties of two domesticated silk films (*Chinese and Thailand B. Mori*) regenerated from formic acid solution, as well as their original fibers, were compared and investigated using dynamic mechanical analysis (DMA) and Fourier transform infrared spectrometry (FTIR). Four relaxation events appeared clearly during the temperature region of 25 °C to 280 °C in DMA curves, and their disorder degree (*f*_dis_) and glass transition temperature (*T*_g_) were predicted using Group Interaction Modeling (GIM). Compared with *Thai* (Thailand) regenerated silks, *Chin* (Chinese) silks possess a lower *T*_g_, higher *f*_dis_, and better elasticity and mechanical strength. As the calcium chloride content in the initial processing solvent increases (1%–6%), the *T*_g_ of the final SF samples gradually decrease, while their *f*_dis_ increase. Besides, SF with more non-crystalline structures shows high plasticity. Two *α*- relaxations in the glass transition region of tan *δ* curve were identified due to the structural transition of silk protein. These findings provide a new perspective for the design of advanced protein biomaterials with different secondary structures, and facilitate a comprehensive understanding of the structure-property relationship of various biopolymers in the future.

## 1. Introduction

Silk is a biopolymer with perfect biocompatibility and tunable biodegradability due to its unique protein compositions and structures [1,2,3,4,5]. In the past few decades, silk has been developed into variable biomaterials including tubes, sponges, hydrogels, fibers and thin films, and combined with various functional nanomaterials to provide unique properties that can be applied to biomedical, electrical, or material engineering [6,7,8,9,10,11].

Generally, different material fabrication methods can affect the multi-step structural transitions and physical properties of silk fibroin materials. For example, Philips et al*.* [12] compared the dissolution of silk fibroin using different ionic liquids, and demonstrated that 1-butyl-3-methylimidazolium chloride, 1-butyl-2,3-dimethylimidazolium, and 1-ethyl-3-methylimidazolium were able to disrupt the hydrogen bonding in silk fibroin fibers. By controlling drying rate, Lu et al. [13] were able to prepare water-insoluble silk films from 9.3 mol/L LiBr aqueous solution. Tian et al*.* [14] added poly epoxy materials, such as formaldehyde, glutaraldehyde, and epoxy compounds into silk fibroin. They suggested that the flexibility of silk materials can be improved through the epoxy compounds, which also acted as crosslinking agents for silk fibroin proteins.

Dynamic Mechanical Analysis (DMA) is one of thermal analysis techniques, which is an advanced technique for measuring the viscoelastic change of polymeric materials during their structural relaxation. [15,16,17,18,19,20] Juan et al*.* [21] investigated the effect of temperature and thermal history on the mechanical properties of native silkworm and spider dragline silks by dynamic mechanical thermal analysis (DMTA). Their results showed that the DMA storage modulus and loss tangent of silk materials depend on their different chemical and physical processing methods. Wang et al*.* [22] also explored the variability of individual as-reeled *A. pernyi* silk fibers using DMTA. They suggested that different polar solvents could affect the tensile properties and structure of silk fibers during the quasi-static tensile tests in ethanol, air, methanol, or water. Porter et al*.* [23,24] assumed that spider silk’s stiffness and strength attributed to the high cohesive energy density of hydrogen bonding, and the toughness attributed to the high energy absorption during post-yield deformation. Furthermore, they found that silk strength was associated with the peculiar molecular and nanoscale structure of its morphology. Kawano et al*.* [25] measured *Nafion* silk films with different types of solvent and cations using DMA in the controlled force mode. Their results demonstrated that silk elasticity decreased with the increase of water, methanol, ethanol, or ethanol/water mixture content in the *Nafion* film, and also decreased with increasing temperature and cation substitutions (Li^+^, Na^+^, K^+^, Cs^+^ and Rb^+^).

Besides, Step-scan Differential Scanning Calorimetry (SSDSC) is a relatively new technique which is another thermal analysis technique under temperature modulation, where the temperature program comprises a periodic succession of short, heating rates, and isothermal steps; thus, the measured heat flow contains contributions which arise from the heat capacity and those due to physical transformations or chemical reactions. The total heat flow can be separated into the reversing and non-reversing components, because the reversing component is only observed on the heating part of the cycle and the non-reversing only on the isothermal. Since both the heat capacity equilibration and DSC equilibration are rapid, the *C*_p_ calculation is said to be independent of kinetic processes [26]. Therefore, through SSDSC, the “reversing heat capacity”, which represents the reversible heat effect of samples within the temperature range of the modulation, such as the specific heat of samples during the glass transition region, can be measured and calculated. Hu et al. [3] used temperature modulated DSC (TMDSC) to eliminate the non-reversing thermal phenomena of the sample and measure the reversing thermal properties of the silk-tropoelastin samples. Sheng et al. [27] characterized the heat capacity, phase contents and transitions of PLA scaffold using SSDSC approach.

*Bombyx Mori* silkworms are domestically raised silkworms that can produce white silk fibers (e.g., from China (*Chin* silk)), or yellow silk fibers (e.g., from Thailand (*Thai* silk)), due to their different geographical and growing environments [28,29]. Derived from *Bombyx mori* cocoons, silk fibroin (SF) is a fibrous protein consisting of repeating glycine-alanine or glycine-serine peptides responsible for beta-sheet crystal structures mixed with amorphous regions [3,4,5,6,7,8,9,10,11]. Different silkworm species have different amino acid compositions and therefore have different crystallinity [5,23,24]. The environmental climate can also affect the mulberry leaves. Various silkworm leaves or foods may lead to differences in their cocoons, such as the color and strength. In our previous work, *Indian Antheraea mylitta*, *Philosamia ricini*, *Antheraea assamensis*, Thailand and Chinese *Bombyx mori* mulberry (*Thai*, *Chin*) silk films have been successfully regenerated from the aqueous solution [30]. Moreover, it was found that Chin and Thai silk fibroin films can be regenerated through a calcium chloride-formic acid (CaCl_2_/FA) solution system [31]. It was demonstrated that Ca^2+^ ions could interact with the silk structure, and change their glass transition temperature, specific heat, and thermal stability [32]. In this work, the DMA technique was, for the first time, used to explore and compare the structure and mechanical property of these two kinds of silk fibers (Thai, Chin), and also combined with SSDSC and FTIR technologies to investigate these properties and transformation mechanism of their protein films regenerated from the FA solution with a changing CaCl_2_ content (1%~6%). In addition, a theory developed by Porter et al. [24] Group Interaction Modeling (GIM), was used to investigate and verified the relationship between the stability and structure of regenerated silk materials during the glass transition temperature region (*T*_g_). This work also explained the impact of CaCl_2_ content to the dynamic mechanical properties of two domesticated silks comprehensively. These comparative studies are important for the design of advanced silk-based materials with tunable structures and properties.

## 2. Results and Discussion

### 2.1. Dynamic Mechanical Analysis of the Degumming Silk Fiber

**Figure 1:**
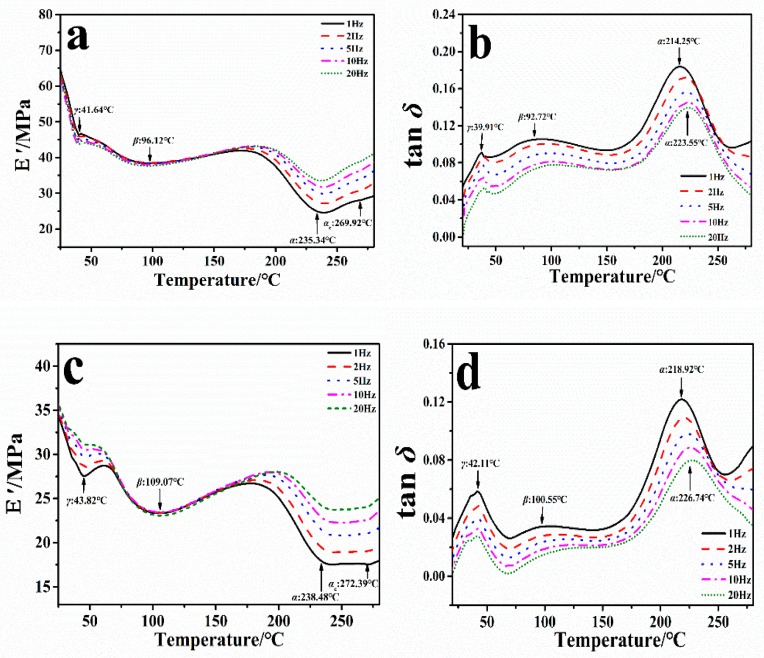
Dynamic mechanical analysis of the storage modulus (*E*′) curves of Chin silk fibroin fibers (**a**) and Thai silk fibroin fibers (**c**); and tan *δ* curves of Chin silk fibroin fibers (**b**) and Thai silk fibroin fibers (**d**) at five different frequencies: 1 Hz (solid line), 2 Hz (dash line), 5 Hz (dot line), 10 Hz (dash dot line), and 20 Hz (short dot line).

Figure 1 shows the storage modulus *E*′ and loss factor tan *δ* curves of CRS and TRS natural fibers with the change of temperature at five frequencies (1 Hz, 2 Hz, 5 Hz, 10 Hz, and 20 Hz), respectively. Four peaks were observed in *E*′ curves of Chin silk fibroin fiber sample under all frequencies (Figure 1a), which were assigned to the protein relaxation of *γ*, *β*, *α*_c_ and *α* at 25 °C to 280 °C, respectively. As the frequency increases from 1 Hz to 20 Hz, the transition peak moves slightly to a higher temperature, since the time of the molecular segment relaxation and movement is inversely proportional to the frequency intensity. According to the equivalent principle of time and temperature, the increased frequency is equivalent to the shortened relaxation time of the material. Therefore, when the material is tested at a higher frequency, the transition peak of the segment movement could move to a higher temperature [33,34]. Different frequencies of 1 Hz to 20 Hz have the same effect of dynamic thermomechanical property on the silk fibroin. Therefore, in the remaining studies, we will only discuss experimental phenomena with a frequency of 1 Hz. In the 1 Hz force-controlled *E*′ curve (solid line), the *γ*-relaxation endear at about 41.64 °C due to the molecular motion of the silk protein side chain and the initial evaporation of free water molecules from silk. The *β*-relaxation at 96.12 °C could not be precisely assigned, but may be related to the molecular motion of silk fibroin after complete evaporation of water, or to the pendant group of the silk polymers (e.g., Ardhyananta et al. [35] has pointed out that the pendant group of the polysiloxanes could affect the thermal and mechanical properties). This can be confirmed by previous findings [36,37] that regenerative silk usually contains 5–10% (*w*/*w*) bound water, which significantly affect the thermal properties of silk. The water content of our samples in this work was around 6 wt.% (Table 1) measured by thermogravimetric analysis (TG), which has been discussed previously [29]. The *α*-relaxation at about 235.34 °C is associated with the glass transition of the silk protein noncrystalline structure due to the segmental motion of the silk protein backbone. Notably, Um et al*.* [38] pointed out that an *α*_c_-relaxation above 260 °C might occur after the *α-*relaxation in silk proteins fabricated from the aqueous solution. In our present work, this relaxation appeared around 269.92 °C for CRS and 272.39 °C for TRS. Besides, during the heating scan, the tan *δ* curves also show clearly three peaks (39.91 °C, 92.72 °C, and 214.25 °C), corresponding to the *γ*, *β*, and *α*-relaxation (Figure 1b). However, the peak of *α*_c_-relaxation did not appear obviously in the tan *δ* curve. The same phenomena can be also found from the TRS fiber sample. For the TRS fiber, three transition events can be observed at 43.82 °C, 109.07 °C, and 238.48 °C in the *E*′ curve at 1 Hz (Figure 1c, solid line), which correspond to *γ*, *β*, *α* relaxations, respectively. The final transition peak at 272.39 °C (Figure 1c) is belonged to *α*_c_-relaxation. Furthermore, in the tan *δ* curve (Figure 1d, solid line), *γ*, *β* and *α-*relaxation peaks were observed at 42.11 °C, 100.55 °C, and 218.92 °C in the range of 25~280 °C, respectively.

Table 2 compared *E*′ and tan *δ* of these two kinds of regenerated silk fibers (CRS and TRS) in the relationship of *γ*, *β*, *α*, and *α*_c_ under the condition of 1Hz frequency. During the *γ*-relaxation, CRS sample has a storage modulus of 18.33 MPa and tan *δ* of 10.45 at 41.64 °C, while TRS sample has a lower storage modulus of 7.48 MPa and a loss factor of 7.21 at 43.82 °C. In the storage modulus *E*′ curve, the peak temperatures of the CRS sample (41.64 °C, 96.12 °C, 235.34 °C, and 269.92 °C) were 2.18~12.95 °C lower than the TRS sample (43.82 °C, 109.07 °C, 238.48 °C, and 272.39 °C) (Table 2). In tan *δ* curve, the peak temperatures of the CRS sample (39.91 °C, 92.72 °C, and 214.25 °C) were also 2.20~4.67 °C lower than those of TRS sample (42.11 °C, 100.55 °C, and 218.2 °C) (Table 2). Moreover, the storage modulus *E*′ of TRS sample under the 1 Hz (7.48 MPa, 11.71 MPa, 23.54 MPa, and 25.73 MPa) were also lower than those of the CRS sample (18.33 MPa, 26.28 MPa, 40.20 MPa and 38.34 MPa) during the *γ*, *β*, *α* and *α*_c_ transitions, respectively (Table 2). These results indicated that *Chin* white silk fiber (CRS) can dehydrate more easily and have more disorder in its structure than the yellow *Thai* TRS fiber, and *Chin* silk molecular chains can be moved more easily when heated. Besides, it will possess a higher degree of viscous deformation, stronger damping, and faster energy dissipation than the TRS sample. Meanwhile, this might also imply that the CRS fiber has more elasticity and stiffness.

Born et al. [39] pointed out that the thermally induced vitreous transition of silk fibroin was proposed to the derive non-cooperative or cooperative movements of the skeleton segments in the non-crystalline or disordered regions of silk structure, when the intermolecular forces pass through a maximum or the intermolecular rigidity tends to zero. The transition condition is known as Born’s elastic instability criterion, which focuses on the stiffness or mobility of the bonds perpendicular to the axis of interaction instead of the bonds along the axis interaction. Quantitatively, Porter’s Group Interaction Modeling (GIM) theory provided a relationship between the properties and structure of polymeric materials, and the expression between structural parameters and *T*_g_ can be presented in Equation (1):

(1) $$T_{g}{}^{c}=0.224y+0.0513\frac{E_{coh}}{N}$$

where the *T*_g_^c^ is the theoretical glass transition temperature at a reference rate of 1 Hz, which can be written in terms of several parameters: (1) the temperature of skeletal mode vibrations, *y*; (2) the cohesive energy, *E_coh_*; and (3) the skeletal degrees of freedom, *N* [24].

The degrees of freedom *N*, in the GIM frame is defined as the number of normal vibration skeletons at the axis of the polymer backbone. For detailed calculation of *E_coh_* for each peptide base, reference can be made to Porter’s work [24]. Wang et al. [22] and Guan et al. [40] studied the cohesive energy *E_coh_* and degree of freedom *N* of each group of China silk from *Bomby Mori cocoon* according to the data in Table 3. *B. mori* silk’ *E_coh_* was calculated as: *E_coh_* = 24.3 (contribution of the peptide base) + 0 × 47.5% (contribution of glycine -H) + 4.5 × 31.7% (Alanine contribution -CH_3_) + 10.8 × 15.8% (Serine -CH_2_-OH contribution) + 35.8 × 5% (tyrosine contribution -CH_2_-Ph-OH) = 29.2 (kJ·mol^−1^).

While the CRS and TRS samples in our study were grown in different regions (China and Thailand) and have different thermal properties [28,29,30], where they all came from *Bombyx Mori* silkworm species. Therefore, we considered that these two kinds of silk have the same cohesive energy *E_coh_* and degree of freedom *N.*

Meanwhile, in our research, the experimental value of *T*_g_ was defined as the temperature from fitted gaussian peak position on the tan *δ* curve during the glass transition, as shown in Figure 1b,d and Table 4. Using the GIM method and the structural parameters in Table 3, we have *E_coh_* = 29.2 kJ·mol^−1^, *N* = 6, and *y* = 241 °C. The value of *y* was common to all structures due to the same average group molecular weight [23]. Therefore, the theoretical value of *T*_g_^c^ of CRS or TRS fiber sample was calculated to 82.4 °C, without considering the contribution from hydrogen-bonds. This theoretical value *T*_g_^c^ was much lower than that of DMA observation (about 218 °C). If one hydrogen bond per peptide group was taken, the calculated result *T*_g_ from Equation (1) would become 157.6 °C, which is close to the lower limit of the silk’s experimental *T*_g_. Guan et al. [40] argued that the molecular structures responsible for the glass transition of silk do not have a singular form, but a probability spectrum with several favored forms, e.g., one or two hydrogen bonds per peptide. Thus, the experimental *T*_g_ temperatures of 214.25 °C for Chin silk fibroin fibers in Figure 1b and of 218.92 °C for Thai silk fibroin fibers in Figure 1d are the results of the averaged hydrogen-bonding density contributed by hydrogen-bonding forms with different probabilities, respectively. Vollrath et al. [23] believed that if one or two hydrogen bonds were adopted, the molecular structure in the silk would have a 70% chance of 2 hydrogen bonds (H-bonds). Hydrogen bond energy took 10 kJ·mol^−1^ as an average of N-H^...^O and N-H^...^N forms, respectively. The higher *T*_g_ implied more hydrogen bonds existed among amide groups of silks, through which highly oriented molecular structure and the number of hydrogen bonds have impact on the cohesive energy directly. In *B. Mori* silk sample, if two hydrogen bonds per peptide were counted, an additional energy of 20 kJ·mol^−1^ would be added to the peptide base value of 29.2 kJ·mol^−1^, which gives the final average *E*_coh_ of 49.2 kJ·mol^−1^ for each characteristic segment. As a result, the theoretical value *T*_g_^c^ of silk is 243.1 °C through Equation (1) calculation, which is close to the upper limit of experimental temperature of *T*_g_ at 214.25 °C in tan *δ* curve and at 235.34 °C in *E*′ curve for CRS fiber sample, while at 218.92 °C in tan *δ* curve, and at 238.48 °C in *E*′ curve for TRS fiber sample.

Tan *δ* curves from DMA can be further used to determine the structural change of regenerated silk. As previously introduced, the order-disorder distribution can avoid the complicated assignments of secondary structures, which could be used to effectively predict the macroscopic properties of silk materials. First, a structural parameter, *f*_dis_, is defined as the degree of structural disorder, which is the molar fraction of the non-crystalline structures that are responsible for glass transition. The degree of structural disorder approximates an averaged structural parameter of heterogeneous nano-structures in a mean-field homogeneous micro and macroscopic morphology. For these two kinds of silk, their *f*_dis_ values obtained from amino acid sequence analysis are listed in Table 4.

Equation (2) from GIM model described the quantitative relationship of cumulative tan *δ* (over the transition temperature range) with the structural parameters of the interactive group, *E_coh_* and *N*. A quick calculation of tan *δ*^c^ using Equation (2) for *B*. *Mori* silk is shown in Table 4, which appeared much greater than the experimental values of tan *δ*. Therefore, for semi-crystalline silks, the degree of structural disorder *f*_dis_ was introduced into the equation, and adapted as a new form, as presented in Equation (3). The function of factor *f*_dis_ is easy to understand as only the motions of the disordered structure could be activated during the glass transition and contribute to the experimentally measured tan *δ*.

The coefficient (2/3) in Equation (3) was used to correct the experimental effect of the uniaxial tensile mode in DMA measurement, because the molecular structures subjected to the static stress of the tensile direction could not be relaxed as the motions along this direction are restrained. As a result, the probability of molecular motions of the overall disordered structure through glass transition was reduced by one dimension, or a factor of 2/3.

(2) $$\tan\delta^{c}=0.0085\times \frac{E_{coh}}{N}\;$$

(3) $$\tan\delta =\frac{2}{3}\times f_{dis}\times 0.0085\frac{E_{coh}}{N}\;$$

Equation (3) opened two avenues: First, it allowed the prediction of the cumulative loss tangent with a known degree of structural disorder. Second, it allowed the calculation of the degree of structural disorder from the theoretical tan *δ*^c^. A quick calculation using GIM framework (Equation (2)) for *B*. *Mori* silk showed that tan *δ*^c^ was in the numerical range of 56–70, which represented the energy dissipation of 100% degrees of structural disorder. The apparent discrepancy between the experimental cumulative tan *δ* and the theoretical tan *δ*^c^ during the glass transition for native *B*. *Mori* silks clearly suggested that crystalline or ordered structure existed in our silk samples. This phenomenon was also mentioned in the work of Porter et al. [41,42,43] The degree of disorder *f*_dis_ for two silk fibers were also calculated individually by using both the number of hydrogen bonds and the cohesive energy from the Equation (3) (Table 4). Simultaneously, apparent discrepancy between the experimental tan *δ* and the theoretical tan *δ*^c^ in glass transition region of silks appeared by using the GIM framework. Guan et al*.* and Porter et al*.* also reported the same phenomenon [21,24]. By comparing the tan *δ* of these two kinds of regenerated *B*. *Mori* silk, the degree of disorder *f*_dis_ was deduced: 0.63 for one H-bond and 0.51 for two H-bonds in the CRS sample, and 0.52 for one H-bond and 0.42 for two H-bonds in the TRS sample, as listed in Table 4. The results showed that the disordered structure of silk fibroin had a significant effect on its glass transition. Additionally, the glass transition temperature (*T*_g_) decreased with the degree of disorder (*f*_dis_) increasing, since tan *δ* is directly associated with the *f*_dis_ as well as the ordered molecular structures in silk.

### 2.2. Structural Transformation of Chin and Thai Silk Protein Films

Glass transition temperature is a characterization temperature at which the chain segment of polymer molecules starts to move, which is related to the flexibility of polymer chains. In the glass transition region, when the semi-crystalline polymer material changes from the solid state to the flowing liquid state, the specific heat of the semi-crystalline polymer material undergoes a discontinuous mutation during the heating process [28,29]. Our previous studies on silk fibroin films by scanning electron microscopy (SEM) [30] and X-ray diffraction (XRD) [33] showed that a high CaCl_2_ concentration can significantly reduce the silk fibril structure and micro-/nanoscale morphology in the silk film. Further, two small diffraction peaks appeared at 20.7° and 24.0° in the XRD curves of low CaCl_2_ concentration sample (e.g., TSF-1.5), which is recognized as the silk I structure. This phenomenon implied that the *β*-sheet crystal content decreases with the increase of calcium chloride concentration, and higher calcium chloride concentrations may disrupt the hydrogen bonds between the silk fibroin molecular chains, which reduces the silk II content and increases the silk I content. Here, we will explore the structure transformation of silk proteins fabricated from CaCl_2_–formic acid solution by using DMA, SSDSC, and FTIR results.

**Figure 2:**
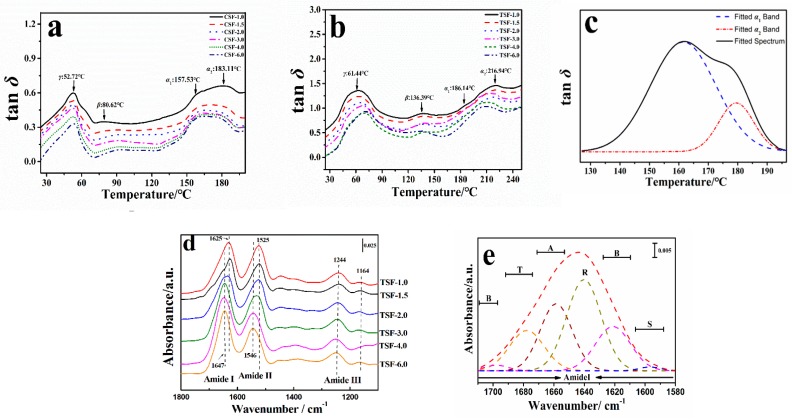
Tan *δ* curves of CSF (**a**) and TSF (**b**) from 1.0% (Solid); 1.5 (Dash); 2.0 (Dot); 3.0 (Dash Dot); 4.0 (Short Dot); 6.0 (Dash Dot Dot) CaCl_2_/FA; (**c**) Curve fitting example of the *T*_g_ region (sample TSF-3.0). The fitted peaks are shown as Dash (*α*_1_ Peak) and Dash Dot Dot (*α*_2_ Peak); (**d**) FTIR absorbance spectra of TSF samples from different CaCl_2_ conctration solutions in the range of 1100–1800 cm^−1^; (**e**) Curve fitting example of the amide I region (sample TSF-3.0) in FTIR spectra. The fitted peaks were shown as short dash-dotted lines, and assigned as side chains (S), *β*-sheets (B), random coils (R), *α*-helixes (A), and turns (T).

In general, the loss factors (tan δ) of silk fibroin membranes CSF-1.0, CSF-1.5, CSF-2.0, CSF-3.0, CSF-4.0, and CSF-6.0 all have three discontinuous events that corresponded to the protein relaxation of γ, β, and α, which have been discussed in the previous section. The γ relaxation peak around 50~60 °C with little shoulders in two side of curve implied the co-events of *γ-*relaxation (protein-water *T*_g_) and the evaporation of mobile H_2_O in this region [21]. The water evaporation completed around 80~140 °C, and the major molecular motion of pure silk fibroin (the *α* relaxation peak of tan *δ *curve) appeared in the range of 150–230 °C (Figure 2). The water content of various silk protein films samples contained around 0.5–3.23% bound water molecules measured by TG in our previous work [30]. Hu et al. [36]. focused on the interaction of the solid silk film with the intermolecular bound water molecules. The results showed that the silk film start to release the water molecules into air at 35 °C. As the temperature increased, more and more water evaporated and the weight of silk film decreased until about 160 °C. Above 160 °C, there is no more intermolecular water in the silk film. Based on these results, the water should have no contribution to *α-*relaxation. In addition, this peak also decreased gradually with the increasing of calcium chloride. To better understand the change during the *T*_g_ region, StepScan differential scanning calorimetry (SSDSC) measurement were also used to determine the heat capacity increment (Δ*C*_p_^s^) of silk samples during the *T*_g_. It demonstrated that the glass transition temperature (*T*_g_^s^) of SF increased with the calcium chloride concentration decreasing, e.g., from 157.30 °C to 176.88 °C with the change of calcium chloride concentration from 6.0% to 1.0%, respectively. The heat capacity increment (Δ*C*_p_^s^) is directly proportional to the average chain mobility of proteins, which reflects the number of freely rotating bonds capable of changing the chain conformation [30]. The Δ*C*_p_^s^ results summarized in Table 5 indicated that a non-crystalline structure exists in all samples and the SF-6.0 protein chains have the highest fraction of the non-crystalline structure, while the average chain mobility and non-crystalline fraction in the SF-1.0 sample are the lowest. Therefore, with the CaCl_2 _concentration increasing, the *T*_g_^s^ decreased while Δ*C*_p_^s^ increased, which indicated that content of non-crystalline structures in SF is increasing with the increase of CaCl_2 _content.

In our previous study [30], we found that the concentration of CaCl_2_/FA could significantly affect the secondary structures of silk. Since DMA technique is more sensitive than the SSDSC technique for the glass transition measurement, two relaxation peaks (*α*_1_ and *α*_2_) can be found in the glass transition region of tan *δ* curve from 125 °C to 195 °C (Figure 2). For the CSF-1.0 sample, the two transition peaks appeared at 157.53 °C (*T*_g-*α*1_) and 177.11 °C (*T*_g-*α*2_), which are associated with *α*_1_ and *α*_2_- relaxation (Figure 2a, solid line), respectively. For the TSF-1.0 sample, the *α*_1_ and *α*_2_-relaxation transitions appeared at 186.14 °C (*T*_g-*α*1_) and 216.94 °C (*T*_g-*α*2_), respectively. To quantify the percentage of these two peaks, the tan *δ* curves were curve fitted using Gaussian peaks in the glass transition region of 125–195 °C.

Figure 2c showed an example of curve fitted tan *δ* curves from the TSF-3.0 sample (dashed lines). Table 5 summarized the fitted percentage of each peak in *T*_g_ region for all silk samples. It shows that the TSF-1.0 film has 54.70% *α*_1_ relaxion at 186.14 °C (*T*_g-*α*1_), and 45.30% *α*_2_-relaxation at 216.94 °C (*T*_g-*α*2_). While the TSF-6.0 sample has 76.65% *α*_1_ relaxion at 182.47 °C and 23.35% *α*_2_ relaxion at 200.82 °C. This suggests that as the concentration of calcium chloride increased, both peaks shifted to lower temperatures (Table 5), but the content of *α*_1_ relaxion increased, while the content of *α*_2_ relaxion decreased. To better understand the secondary structures of our materials, a FTIR deconvolution method was performed to quantify the percentage of the secondary structures in all silk samples [28]. Figure 2d showed the main characteristics of protein structures of all six silk films in the FTIR spectra. With the concentration of CaCl_2_ increasing, the center of the absorption band in Amide I region shifted gradually from 1625 cm^−1^ (beta-sheet structure, TSF-1.0) to 1647 cm^−1^ (random coil structure, TSF-6.0), while the peak in Amide II region also shifted gradually from 1525 cm^−1^ (TSF-1.0) to 1546 cm^-1^ (TSF-6.0). Moreover, for the Amide-III and FTIR fingerprinting regions, Thai silk sample showed same characteristic peaks and two obvious peaks at 1244 cm^−1^ and 1164 cm^−1^ [30]. Figure 2e shows an example of the Amide I region of TSF-3.0 sample with the fitted FTIR peaks shown as dashed lines. The peak positions and their related secondary structures were assigned as side chains (S), *β*-sheets (B), random coil (R), *α*-helix (A), and turns (T) [30]. We found that the content of helix structures in TSF samples could increase significantly by increasing the concentration of CaCl_2_. For example, with the increase of CaCl_2_ concentration, the percentage of *β*-sheet structures gradually decreased from 26.60% in TSF-1.0 film to 7.07% in the TSF-6.0 sample, while the percentage of random coils decreased from 56.07% to 46.43%, while *α*-helixes content increased from 8.59% to 28.39%, and the percentage of turns also increased from 7.11% to 17.74%. The similar trend of *α*-relaxation temperatures and the content of secondary structures in the silk samples were also observed in CSF samples. Table 6 summarized the percentage of secondary structures for each CSF and TSF sample. These results implied that *α*_1_ and *α*_2_- relaxation events in tan *δ* curve are associated with the change of protein secondary structures. Um et al*.* [38] found that there is *α*_c_-relaxation after the major *α-*relaxation in water-regenerated silk materials, which is related to the cooling crystallization of silk proteins. In our silk samples regenerated from CaCl_2_/FA system, with the decrease of *β*-sheets in the silk films, the *α*_1_ content increased, while *α*_2_ percentage decreased, and both of the *α*_1_ and *α*_2_ peaks shifted to a lower temperature. Therefore, we can claim that random coils or other non-crystalline structures may be contributed to the transition of *α*_1_-relaxation which was equaled to the *α*-relaxation, while the change of *α*-helix structure may be related to *α*_2_-relaxation which was the *α*_c_-relaxation found previously [38].

Similarly, DMA experiment also showed that *T*_g-*α*1_ and *T*_g-*α*2_ of the CSF samples were lower than those of the TSF samples (Figure 2 and Table 5) at the same calcium chloride content. Both FTIR and DMA results indicate that lower *β*-sheet structures and higher non-crystalline structures can be obtained in silk films regenerated from the solution with higher calcium chloride concentration.

Base on the above work, the relationship between degree of disorder *f*_dis_ and the *T*_g_ were discussed and the influence of calcium chloride on structure of these silk samples was further explored in this section. It was supposed that the amount of hydrogen bonding effects of calcium chloride on different silk fibroin films were identical (H-bonds = 1; *N* = 6; *E*_coh_ = 39.2 kJ·mol^−1^, *y* = 241 °C), the degree of disorder *f*_dis-*α*1_ and *f*_dis-*α*2_ of two kinds of regenerated silk fibroin were calculated from GIM Equation (3) and is shown in Table 5, as well as the tan *δ*_-*α*1_, tan *δ*_-*α*2_, *T*_g-*α*1_, and *T*_g-*α*2_ from DMA test. The increasing trend of *f*_dis-*α*1_ and *f*_dis-*α*2_ indicated that more concentration of calcium chloride could induce the *β*-sheet transform into the random coil in the secondary structure of silk fibroin and more non-crystalline structure form. Simultaneously, in the same concentration of calcium chloride, CSF samples showed more disorder degree than that of TSF samples, e.g., 0.20 for CSF-1.0 sample, 0.10 for TSF-1.0 sample at *α*_1_-relaxation. The results suggested that more *β*-sheet and crystalline structures in TSF sample than those in CSF sample.

### 2.3. Stress-Strain Study of CSF and TSF 

**Figure 3:**
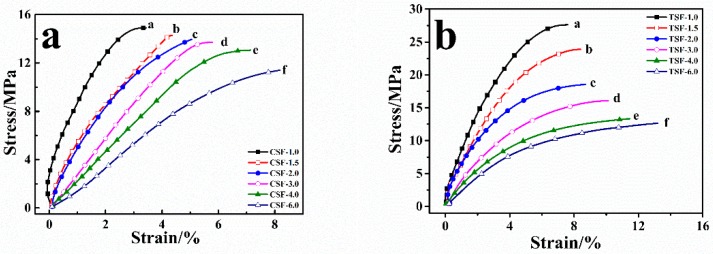
The stress-strain curves of CSF (**a**) and TSF (**b**) samples regenerated from 1.0% (solid square), 1.5% (hollow square), 2.0% (solid circle), 3.0% (hollow circle), 4.0% (solid triangle), and 6.0% (hollow triangle) CaCl_2_/FA solutions.

The stress-strain curve gives information about the Young modulus (slope at the origin), yield point, break point, and recovery behavior of polymeric films. Meanwhile, the stress-strain curve analysis can also provide information on polymer structure, degree of crosslinking, degree of crystallization, processing conditions, and viscoelastic properties of polymer [44,45,46]. The stress-strain curve of the CSF sample (Figure 3a) shows that as the calcium chloride concentration increases from 1.0 to 6.0 mass%, the initial slope decreases from 7.86 to 1.83 and its stress decreases from 14.91 to 11.93 MPa at the yield point, respectively. In addition, under the stress of 10 MPa, their strains were 1.21%, 2.15%, 2.47%, 3.39%, 4.17%, and 5.88%, respectively (Table 7). Compared with the trend of change in CSF samples under the initial slope and yield stress, the trend of change in TSF samples is the same (Figure 3b). These results reveal that increasing the content of calcium chloride can increase the elongation of the sample, while its elasticity and stiffness decrease due to the formation of dominated random coils and other non-crystalline structures in silk samples [33]. Besides, with the same concentration of calcium chloride and the same stress loaded on the sample, the initial slope of CSF sample was higher than that of the TSF sample. For example, the initial slope of the CSF-6.0 sample is 1.83 under the 10 MPa, which was higher than the initial slope of 1.52 from the TSF-6.0 sample.

Similarly, the strain of the TSF-6.0 sample was 6.96%, which was greater than 5.88% of the CSF-6.0 sample. And the yield stress of CSF-6.0 sample was 11.43 MPa, which was higher than 8.93 MPa of the TSF-6.0 sample. These results indicate that the CSF sample has better elasticity than the TSF sample, while the TSF sample has better plasticity than the CSF sample. These results also imply that the average chain mobility of silk films in TSF samples is higher than that in CSF samples. Therefore, at the same CaCl_2_ concentration, the TSF sample would contain more non-crystalline structures than the CSF sample, which has been proved by FTIR structural analysis in Table 6.

## 3. Materials and Methods

### 3.1. Materials and Preparation

The Chinese (Dandong Qiyue Trade co., LTD.) and Thailand *Bombyx mori* silk cocoons (Queen Sericulture Center, Nakornratchasima, Thailand) were first degummed into Chin regenerated silk fibroin fibers (CRS) and Thai regenerated silk fibroin fibers (TRS) according to a previously reported procedure [47]. Different amounts of calcium chloride (AR, purity 96%, West Gansu Chemical Plant, Shantou, Guangdong) were mixed with formic acid (AR, purity 88%, West Gansu Chemical Plant, Shantou, Guangdong) to form 1.0, 1.5, 2.0, 3.0, 4.0, and 6.0 mass% CaCl_2_/FA solutions. Then, the degummed silk fibroin (SF) fibers were quickly dissolved into these CaCl_2_/FA solutions at room temperature. The final solutions were immediately cast onto a Teflon mold (50 mm × 20 mm × 10 mm) and dried at room temperature to form SF films. After being washed (30 mins in running water) and vacuum dried to fully remove CaCl_2_/FA solvents, the final Chin SF (CSF) and Thai SF (TSF) films were obtained. CSF and TSF samples were named with the numbers (−1.0, −1.5, −2.0, −3.0, −4.0, and −6.0) after the sample names to indicate the initial concentration of CaCl_2_ (1.0, 1.5, 2.0, 3.0, 4.0, and 6.0 mass%) in the solution.

### 3.2. Thermal and Mechanical Analyses

Sample with a dimension of 5.0 × 2.0 × 1.0mm^3^ was subjected to the mechanical analysis by using a Dynamic Mechanical Analyzer (Perkin-Elmer Diamond DMA, Waltham, MA, USA). Experiment was proceeded under a temperature range from 25 °C to 280 °C with a heating rate of 2 °C min^−1^ and at 1, 2, 5, 10, and 20 Hz frequencies simultaneously. The glass transition temperature (*T*_g_) of sample was taken from the temperature at which the maximum peak of the tangent *δ* was exhibited. Furthermore, at the atmosphere temperature (25 °C), the stress-strain property of silk fibroin film was tested by DMA in a tensile controlled model. The tensile force increased from 5 mN to 2000 mN at a lifting speed of 50 mN min^−1^ until the sample was broken. For all experiments, at least three samples were measured under the same test conditions to check the consistency of the experimental results. Dynamic mechanical analysis (DMA) refers to the technique of measuring the dynamic modulus and mechanical loss of a specimen and its relation to temperature or frequency under programmed temperature and alternating stress. In general, a periodically varying (usually refers to the sine) force was applied to the sample to produce periodic changes in the stress. The sample will have a corresponding deformation behavior of the stress; thus, the mechanical modulus of the sample can be determined by the stress and deformation. For example, if a shear stress is applied, a shear *G* can be obtained, and if a type of stress is applied in tension or bending, Young’s Modulus *E* can be obtained. DMA measurement modes include tension, compression, bending, single-cantilever, shear, and reverse. In most cases, the specimen undergoes a periodical variation of the mechanical vibration stress, which causes the corresponding vibrational strain. However, the specimen does not always respond instantaneously to the changing stress and the lags behind for a certain period of time. This mainly depends on the viscoelasticity of the specimen and the phase shift between applied stress and deformation. Therefore, sample modulus consists of both real and imaginary parts. The real part describes the sample response as the periodic stress, which is a measurement of the elasticity of the sample, called the storage modulus. The imaginary part describes the response phase shifted by 90°, which is a measurement of the mechanical energy converted to heat, called the loss modulus. The ratio of loss modulus (*E*″) and storage modulus (*E′*) is called the loss factor, the phase shift tangent *δ* (tan *δ*), which is the express of sample damping performance. The storage modulus *E*′ is proportional to the mechanical energy stored in the specimen during stress, while the loss modulus *E*″ represents the energy dissipated in the specimen during stress. The larger the loss modulus is, the more viscous the specimen is, and the stronger the damping of specimen is. The tan *δ* is independent of the geometry, therefore it can be accurately measured even if the geometry of the sample is not regular. In dynamic mechanical analysis, the force amplitude *F*_A_ and the displacement amplitude *L*_A_ are used to calculate the modulus of samples. For example, the tensile modulus or elastic modulus of the experiment *E*′ can be obtained through the formula:

(4) $$E^{\prime }=\frac{\sigma }{\varepsilon }=\frac{F_{A}}{L_{A}}\frac{L_{0}}{A}=\frac{F_{A}}{L_{A}}g\;$$

*σ* = *F*_A_/*A* (5)

*ε* = *L*_0_/*L*_A_ (6)

*g* = *L*_0_/*A* (7)

where *σ* and *ε* are the stress and strain of the sample, respectively; *F*_A_ and *L*_A_ are the force amplitude and the displacement amplitude, respectively; *L*_0_ is the original length of the sample; *A* is the unit area of the sample; *g* is the geometry efficiency; and *F*_A_/*L*_A_ is the rigidity of the material [48].

Generally, materials with low storage modulus *E*′ implies it will deform easily when applying load on it; and the tan *δ* represents the viscoelasticity of materials during the loading cycle. A high value of tan *δ* suggests a high degree of energy dissipation and a high degree of viscous deformation for this material.

Besides, the dried CRS and TRS samples were encapsulated in Al pans and heated in a differential scanning calorimeter under Step-Scan modulated (SSDSC, Diamond DSC, Perkin-Elmer, USA) at a heating rate of 3 °C/min^−1^ with a 3 °C step and isothermal time of 2 min with 25 mL min^−1^ purged dry nitrogen gas, and equipped with a refrigerated cooling system. Each sample was about 5 mg. 

### 3.3. Fourier Transform Infrared Spectrometry

Fourier transform infrared spectra (FTIR) of silk protein film sample was obtained using a FTIR spectrometer (Nicolet-NEXUS 670, Nicolet, Madison, WI, USA), equipped with a deuterated triglycine sulfate detector and a multiple-reflection, horizontal MIRacle ATR attachment (OMNIT, using a Ge crystal, Madison, WI, USA). Spectra were recorded in the wavenumber range of 1800 to 1100 cm^−1^ with a resolution of 4 cm^−1^, and 64 scans were applied for each measurement. Fourier self-deconvolution (FSD) of the IR spectra covering the Amide I region (1595–1705 cm^−1^) was performed by the Nicolet Omnic software. Deconvolution was performed using Gauss line shape with a half-bandwidth of 25 cm^−1^ and a noise reduction factor of 0.3. FSD spectra were then curve-fitted by Gaussian peaks to measure the relative areas in the Amide I region.

### 3.4. Thermogravimetric Analysis

Thermogravimetric (TG) analysis (PerkinElmer Pyris 1, Waltham, MA, USA) was used to measure the change in the mass of the silk samples during temperature increase. The TG curves were obtained under a nitrogen atmosphere with a gas flow of 50 mL·min^−1^. Samples of about 2–3 mg were heated from 30 to 600 °C with a heating rate of 10 °C·min^−1^. The mass change percentages during heating were recorded.

## 4. Conclusions

Dynamic Mechanical Analysis (DMA) analysis is more sensitive than Differential Scanning Calorimetry (DSC), as short-range chain motion changes are easier to detect than heat capacity changes during the phase transitions of biopolymer materials. The various structures and mechanical properties of two kinds of domesticated silk fibroin (SF) films (Chinese and Thailand *B. Mori*) regenerated from formic acid-CaCl_2_ solutions were investigated using DMA. Our study showed that by using the GIM model, the disorder degree (*f*_dis_) of silk samples can be inferred from the cohesive energy (*E*_coh_), the skeletal degree of freedom (*N*), and the loss factor (tan *δ*) at the glass transition region. Four disordered phase relaxation events were explored by DMA technique, with a focus on the glass transition and the degree of structural disorder. Our results illustrated that there are nearly two hydrogen bonds formed in each peptide group in the *Bombyx Mori* silk fibers. With the increase of calcium chloride concentration in the SF sample, the *T*_g_ of silk material decreases, which implied that the sample could contain more non-crystalline structures, such as random coils and helix. *α*_1_ and *α*_2_-relaxation events in DMA curves are both associated with the silk secondary structures. The random coils as well as other non-crystalline structures may be attributed to the change of *α*_1_-relaxation, while *α*_2_-relaxation could be directly associated to the *α*-helix to *β*-sheet transition. Moreover, at the same calcium chloride concentration, the CSF sample is more disordered than the TSF sample, which suggests that there are more *β*-sheet in the TSF sample than in the CSF sample. It also showed that the elasticity of the TSF sample is lower than that of the CSF sample, while their ductility is the opposite. Besides, SF samples prepared with lower concentrations of calcium chloride have higher elasticity, while SF samples prepared with high concentrations have better ductility. The effects of calcium chloride concentrations on the structure and mechanical properties of regenerated silk fibroin films were further investigated by comparing GIM theoretical model calculations with experimental results. These results provide us a new way to understand the structural changes and mechanical properties of different domesticated silk regenerated from acid-based solution system, which would be critical for engineering applications of silk materials. These results provide us with a new way to understand the structural changes and mechanical properties of different domesticated silk regenerated from acid-based solution system, which would be critical for engineering applications of silk materials in the future.

## Figures and Tables

**Table 1:** The water content of different SF film samples by Thermogravimetric Analysis (TG) *.

Sample	Water Content/%	Sample	Water Content/%
**CRS**	9.70	TRS	7.26
**CSF-1.0**	3.23	TSF-1.0	1.26
**CSF-1.5**	2.97	TSF-1.5	1.04
**CSF-2.0**	2.05	TSF-2.0	1.10
**CSF-3.0**	1.79	TSF-3.0	1.20
**CSF-4.0**	1.35	TSF-4.0	0.52
**CSF-6.0**	1.16	TSF-6.0	0.51

* All of the numbers have an error bar of ± 3%.

**Table 2:** The experimental parameters of degummed Chinese (CRS) and Thailand (TRS) *B. Mori* silk fibers obtained from DMA analysis *

Sample	CRS/TRS			
Attribution	*γ*	*β*	*α*	*α*_c_
***E*′/MPa**	18.33/7.48	26.28/11.71	40.20/23.54	38.34/25.73
**Tan *δ***	10.45/7.21	6.24/4.72	23.49/19.36	N/A
***T_E_*_′_/°C**	41.64/43.28	96.12/109.07	235.34/238.48	269.92/272.39
***T_t_*_an*δ*_/°C**	39.91/42.11	92.72/100.55	214.25/218.92	N/A
**Δ*T_E_*_′_/°C**	2.18	12.95	3.14	2.47
**Δ*T_t_*_an*δ*_/°C**	2.20	7.83	4.67	N/A

* *E*′ and tan *δ* represent the storage modulus and integral loss factor of CRS and TRS samples at 1 Hz frequency respectively in DMA tensile mode. *T_E_*_′_ and *T_t_*_an*δ*_ represent the peak temperatures for the storage modulus and the loss factor curves, which are corresponding to the protein relaxation of *γ*, *β*, *α* and *α*_c_. Δ*T_E_*_′_ and Δ*T*_tan*δ*_ represent the peak temperature differences between TRS and CRS samples at *γ*, *β*, or *α*-relaxation regions. The *E*′ and Tan *δ* values have an error bar of ±0.3, the *T_E_*_′_ and *T_t_*_an*δ*_ values have an error bar of ±0.5 °C.

**Table 3:** Calculated GIM parameters based on peptide group contributions and amino acid (AA) sequences for *B Mori* silks [24].

Peptide	Structure	*E_coh_*/kJ·mol^−1^ (without H-Bond)	Degrees of Freedom *N*	Molar Fraction as Counted in AA Sequence *B Mori*
**Peptidebase(-R group)**	-C-CO-NH-	24.3	6	0
**Glycine(G)**	-H	0	0	47.5%
**Alanine(A)**	-CH_3_	4.5	2	31.7%
**Serine(S)**	-CH_2_-OH	10.8	3	15.8%
**Glutamine(Q)**	-CH_2_-CH_2_-CO-NH_2_	28.8	5	0
**Tyrosine(Y)**	-CH_2_-Ph-OH	35.8	4	5%
**Leucine(L)**	-CH_2_-C(CH3)_2_	18	4	0
**Arginine(R)**	-CH_2_-CH_2_-CH_2_-NH-C(NH_2_)_2_	45	7	0
**Average**	*B. Mori*	29.2	6	/

**Table 4:** GIM parameters used for *T*_g_ and the degree of structural disorder *f*_dis_ calculations b*

Sample	Group	H-Bonds	*E_coh_*/kJ·mol^−1^	*N*	*T*_g_^c^/°C	*T*_g_/°C	Tan *δ*^c^	Tan *δ*	*f*_dis_
**Chin**	G_0.475_A_0.317_S_0.158_Y_0.005_	1 2	39.2 49.2	6 6	157.6 243.1	214.25	56 70	23.49	0.63 0.51
**Thai**	G_0.475_A_0.317_S_0.158_Y_0.005_	1 2	39.2 49.2	6 6	157.6 243.1	218.92	56 70	19.36	0.52 0.42

* The number of H-bonds per peptide group is 1 or 2. Cohesive energy *E_coh_* is the sum of energy from hydrogen bonds and the peptide base. *N* is the degrees of freedom. *T*_g_^c^ is the theoretical glass transition temperature calculated from Equation (1), in which *y* is set as 241 °C for all cases. *T*_g_ represents the experimental glass transition temperature from tan *δ* curve at *α* relaxation. The theoretical Tan *δ*^c^ was calculated from Equation (2), which represents the energy dissipation for 100% degrees of structural disorder. Tan *δ* is the integral loss factor at *α* relaxation in DMA curve, and *f*_dis_ is the predicted degree of structural disorder by using Equation (3).

**Table 5:** SSDSC and DMA analysis of CSF and TSF samples in their glass transition region [30] *.

Sample	[CaCl_2_]/wt %	*T_g_*^s^/°C	Δ*C*_p_/J·g^−1^·°C^−1^	*T*_g-*α*1_/°C	Content in *α*_1_ Region/%	Tan *δ*_-*α*1_	*f*_dis-*α*1_	*T*_g-*α*2_/°C	Content in *α*_2_ Region/%	Tan *δ*_-*α*2_	*f*_dis-*α*2_
**CSF**	1.0	176.88	0.1667	157.53	32.87	6.65	0.18	177.11	67.13	7.52	0.20
	1.5	172.65	0.1721	155.49	52.56	7.15	0.19	171.47	47.44	7.92	0.21
	2.0	169.04	0.1890	155.16	53.95	7.89	0.21	168.32	46.05	8.91	0.24
	3.0	167.42	0.1998	154.87	71.53	9.89	0.27	165.51	28.47	9.25	0.25
	4.0	163.21	0.2157	154.39	73.54	11.57	0.3	163.93	26.46	10.37	0.27
	6.0	157.30	0.2204	153.92	79.99	12.44	0.34	162.36	20.01	11.03	0.30
**TSF**	1.0	181.54	0.1641	186.14	54.70	4.34	0.12	216.94	45.30	4.02	0.10
	1.5	177.08	0.1643	184.54	65.51	5.73	0.15	212.01	34.49	4.97	0.13
	2.0	175.94	0.1644	184.31	71.14	6.52	0.18	209.27	28.86	5.56	0.14
	3.0	173.42	0.1972	183.69	75.24	7.22	0.19	206.45	24.76	6.25	0.16
	4.0	164.01	0.2136	183.08	75.72	8.93	0.23	204.85	24.28	7.67	0.20
	6.0	159.20	0.2137	182.47	76.65	10.17	0.26	200.82	23.35	8.93	0.23

* [CaCl_2_] is the concentration of calcium chloride in the solution. *T_g_*^s^ is the glass transition temperature of SF measured by SSDSC. *T*_g-*α*1_ and *T*_g-*α*2_ represent the peak temperatures of *α*_1_-relaxtion and *α*_2_-relaxtion from the tan *δ* curves, respectively. Their content values were obtained by fitting the tan *δ* curve in *α*_1_ and *α*_2_-relaxtion regions using Gaussian peaks. Tan *δ_-α_*_1_ and Tan *δ*_-*α*2_ are the integral loss factor at *α* relaxations. *f*_dis-*α*1_ and *f*_dis-*α*2_ are the predicted degree of structural disorder at *α*_1_-relaxtion and *α*_2_-relaxtion by the GIM model, respectively. The *T_g_*^s^, *T*_g-*α*1_, and *T*_g-*α*2_ have an error bar of ±0.5 °C. The Content in *α*_1_ region and Content in *α*_2_ region have an error bar of ±3%. The Tan *δ*_-*α*1_, Tan *δ*_-*α*2_, *f*_dis-*α*1_ and *f*_dis-*α*2_ have an error bar of ±0.05.

**Table 6:** Percentage of secondary structures in CSF and TSF samples obtained by FTIR structural analysis [28].

	CSF					TSF				
[CaCl_2_] (wt %)	*β*-Sheet/%	Turns/%	Side Chains%	*α*-Helix/%	Random Coils/%	*β*-Sheet/%	Turns/%	Side Chains/%	*α*-Helix/%	Random Coils/%
1.0	23.32	6.03	1.33	10.07	59.25	26.60	7.11	1.63	8.59	56.07
1.5	20.25	7.59	1.07	11.39	59.70	24.76	8.02	1.15	9.96	56.11
2.0	13.56	12.05	0.91	13.78	59.70	14.70	14.31	1.19	12.54	57.26
3.0	8.09	14.13	0.85	18.79	58.14	10.92	16.61	0.94	18.22	53.31
4.0	8.17	15.41	0.63	25.66	50.13	8.25	17.05	0.79	21.83	52.08
6.0	6.83	15.89	0.57	30.26	46.45	7.07	17.74	0.37	28.39	46.43

**Table 7:** Mechanical properties of CSF and TSF samples measured by DMA *.

	CSF			TSF		
[CaCl_2_]/wt %	Initial Slope	Yield Stress/MPa	Strain/%	Initial Slope	Yield Stress/MPa	Strain/%
1.0	7.86 ± 0.10	14.91 ± 1.57	1.21 ± 0.08	7.37 ± 0.24	14.28 ± 1.29	1.86 ± 0.07
1.5	7.11 ± 0.32	14.30 ± 1.38	2.15 ± 0.15	6.94 ± 0.19	13.33 ± 1.37	2.27 ± 0.10
2.0	5.48 ± 0.55	13.98 ± 1.55	2.47 ± 0.13	3.57 ± 0.36	12.45 ± 1.56	2.50 ± 0.05
3.0	2.96 ± 0.37	13.66 ± 1.29	3.39 ± 0.16	2.70 ± 0.27	11.20 ± 1.28	3.93 ± 0.12
4.0	2.16 ± 0.86	12.97 ± 1.37	4.17 ± 0.09	1.80 ± 0.58	10.17 ± 1.35	5.45 ± 0.27
6.0	1.83 ± 0.27	11.43 ± 0.98	5.88 ± 0.17	1.52 ± 0.49	8.93 ± 1.88	6.96 ± 0.16

* [CaCl_2_] is the concentration of calcium chloride in the formic acid solution system. The initial slope is the ratio of stress to strain, which represents the elasticity of SF sample. The yield stress is the stress at the yield point in stress-strain curve. The strain value is the material elongation ratio under the stress force of 10 MPa. Every sample have to do five experiment times. Their errors or deviations were shown in each column after symbol ‘±’.
